# The diagnostic performance of CA-125 for the detection of ovarian cancer in women from different ethnic groups: a cohort study of English primary care data

**DOI:** 10.1186/s13048-024-01490-5

**Published:** 2024-08-26

**Authors:** Melissa Barlow, Liz Down, Luke T. A. Mounce, Garth Funston, Samuel W. D. Merriel, Jessica Watson, Gary Abel, Lucy Kirkland, Tanimola Martins, Sarah E. R. Bailey

**Affiliations:** 1 Department of Health and Community Sciences, University of Exeter, St Lukes Campus, Heavitree Road, Exeter, EX1 2LU UK; 2 Centre for Cancer Screening, Prevention and Early Diagnosis, Wolfson Institute of Population Health, Queen Mary University of London, London, EC1M 6BQ UK; 3 Centre for Primary Care & Health Services Research, University of Manchester, Oxford Road, Manchester, M13 9PL UK; 4 Centre for Academic Primary Care (CAPC), Population Health Sciences, Bristol Medical School, University of Bristol, Canynge Hall, 39 Whatley Road, Bristol, BS8 2PS UK

**Keywords:** Ovarian cancer, Diagnosis, CA-125, Ethnic groups, Primary care

## Abstract

**Background:**

CA-125 testing is a recommended first line investigation for women presenting with possible symptoms of ovarian cancer in English primary care, to help determine whether further investigation for ovarian cancer is needed. It is currently not known how well the CA-125 test performs in ovarian cancer detection for patients from different ethnic groups.

**Methods:**

A retrospective cohort study utilising English primary care data linked to the national cancer registry was undertaken. Women aged ≥ 40 years with a CA-125 test between 2010 and 2017 were included. Logistic regression predicted one-year ovarian cancer incidence by ethnicity, adjusting for age, deprivation status, and comorbidity score. The estimated incidence of ovarian cancer by CA-125 level was modelled for each ethnic group using restricted cubic splines.

**Results:**

The diagnostic performance of CA-125 differed for women from different ethnicities. In an unadjusted analysis, predicted CA-125 levels for Asian and Black women were higher than White women at corresponding probabilities of ovarian cancer. The higher PPVs for White women compared to Asian or Black women were eliminated by inclusion of covariates.

**Conclusion:**

The introduction of ethnicity-specific thresholds may increase the specificity and PPVs of CA-125 in ovarian cancer detection at the expense of sensitivity, particularly for Asian and Black women. As such, we cannot recommend the use of ethnicity-specific thresholds for CA-125.

**Supplementary Information:**

The online version contains supplementary material available at 10.1186/s13048-024-01490-5.

## Background

In the UK, ovarian cancer is the sixth most common cancer in females, with an estimated 7500 new cases and over 4000 deaths annually [1]. It has the worst prognosis of all gynaecological malignancies with fewer than half of those with ovarian cancer being diagnosed at an early stage (I-II) [1]. The NHS Long Term plan has set a target that by 2028, 75% of people with cancer will be diagnosed at an early stage (I-II) [2]. Recent guidance for the 28-day Faster Diagnosis Pathway for Gynaecological Cancer detailed the secondary care pathway for suspected ovarian cancer [3]. In primary care, the National Institute for Health and care Excellence (NICE) guidance for suspected cancer recommend urgent referral to gynaecological services in the presence of ascites and/or pelvic/abdominal mass [4]. Other clinical features of suspected ovarian cancer include bloating, abdominal and pelvic pain, early satiety and increased urinary frequency. [4]. These features are common in primary care and have positive predictive values below the 3% threshold recommended in NICE for specialist investigation [5]. The low positive predictive values of these symptoms can make clinical decision-making challenging.

Levels of CA-125, a protein biomarker, are often elevated in patients with ovarian cancer. General practitioners (GPs) can offer women with suspected ovarian cancer symptoms a CA-125 test, which measures levels of CA-125 in the blood. NICE NG12 advocates further investigation for CA-125 levels of ≥ 35U/ml and no further investigations for ovarian cancer in patients whose CA-125 level is less than 35U/ml [4].

The diagnostic performance of CA-125 for the detection of ovarian cancer in primary care has been extensively studied [6, 7], although little is known about its performance in women from diverse ethnic backgrounds. In a recent modelling study that predicted ovarian cancer using a patient’s CA-125 result and other risk information, ethnicity (White or Non-White) was associated with cancer risk in multivariable analysis and included in the final predication model [8], although the contribution of ethnicity and its relationship to CA-125 level was not explored further. Previous reports indicate CA-125 levels differ by ethnicity in both women with [9] and without ovarian cancer [10, 11], although there have been no studies in primary care. Therefore, identifying whether there are differences in CA-125 levels in women of different ethnic groups who present to primary care, and in ovarian cancer incidence following a CA-125 test, will help determine whether CA-125 performs equally well for women from all ethnic groups. This may refine the identification of women who could benefit from further investigation for ovarian cancer and may also help to reduce health inequalities.

This study examined the association between patient ethnicity and ovarian cancer diagnosis following a CA-125 test.

## Methods

### Data sources and patients

This cohort study used electronic health records from the Clinical Practice Research Datalink (CPRD) Aurum dataset [12], which contains information on patient demographics (including ethnicity), diagnoses, blood test results, and prescriptions in primary care. As of May 2022, the CPRD Aurum dataset contained 13,300,067 currently registered patients, representing 19.8% of the UK population [13]. Anonymised primary care records were linked to the Hospital Episode Statistics Admitted Patient Care (HES APC) dataset [14] to gather ethnicity data where this was missing from the CPRD dataset.

Included patients were women aged 40 years and over who were registered with a GP practice in England contributing to CPRD during the study period 2010–2017. Participants had an ethnicity record and a valid CA-125 test result recorded during the study period. Ethnicity was categorised into five broad ethnic groups, commonly used in UK statistics (White, Asian, Black, Mixed, and Other). An algorithm was used to determine patient ethnicity, which closely follows that used in previous ethnicity research in the UK [15–17]. This is detailed in a recent publication [18]. Women with any recorded cancer (except for non-melanoma skin cancer) before the first CA-125 test date, or women who died within one year of the test date with no ovarian cancer diagnosis, were excluded from the analysis.

Incident diagnoses of ovarian cancer in the year following the patients’ first CA-125 test in the study period were identified from the English National Cancer Registration and Analysis Service (NCRAS) cancer registration dataset [19]. Patients with no record of an ovarian cancer diagnosis in the year following a CA-125 test were considered as not having been diagnosed with cancer, in line with previous work [6].

### Statistical analysis

Multi-level logistic regression, clustering patients within GP practices, assessed whether the predictive value of an abnormal CA-125 test result for ovarian cancer varied across different ethnic groups. CA-125 was categorised as normal (< 35U/ml) or high (≥ 35U/ml). A secondary analysis examined the association of a high CA-125 result with the diagnosis of ovarian cancer at an advanced stage (III-IV) within one year of the test date. Both models included an interaction term to assess whether the association of a high CA-125 result and an incident diagnosis of ovarian cancer differed by ethnic group. For both analyses, the marginal distributions of the models were used to obtain estimated cancer incidences across patient groups adjusted for all covariates.

Covariates included for analysis were: age group in ten-year bands (40–49 years, 50–59 years, 60–69 years, 70–79 years, ≥ 80 years), quintile of area-based deprivation score (IMD 2015 [20]), tertile of Cambridge Multimorbidity Score (CMS) [21], and year of blood test. As different ovarian cancer histologies have different propensities to raising the CA-125 levels, the secondary stage analysis also included ovarian cancer histology as a categorical covariate (serous, mucinous, endometroid, clear cell, other epithelial, unknown epithelial, and non-epithelial. ICD-O morphology and behaviour codes used to define histology can be found in Supplementary 1).

Logistic regression predicted the odds of an ovarian cancer diagnosis (a binary outcome) by CA-125 level as a continuous variable (up to a value of 1000U/mL), which was subsequently converted into predicted ovarian cancer incidence rates, as described previously [6]. Here, the analysis was stratified by ethnic group to estimate ethnic variation in the one-year incidence of an ovarian cancer diagnosis by CA-125 level (normal or high) and identify the threshold above which predicted ovarian cancer incidence is 3% or higher. As CA-125 has greater accuracy in women aged ≥ 50 years [6], most ovarian cancers occur in this group, and NICE guidelines recommend CA-125 testing in women with non-specific symptoms of ovarian cancer particularly if they are aged ≥ 50 years, these analyses were reported for women aged ≥ 50 years only, with the results for women aged ≥ 40 years included in the supplementary material. This also controlled for differences in age distributions (the average age of ethnic minority groups presenting to primary care is lower than the White majority [18]), and menopausal status (previous research stated the cut-point for defining menopausal status for CA-125 testing to be 50 years [22]). CA-125 was highly skewed, so it was log transformed prior to analysis and centred on the value of 3 as the closest integer to the mean. The relationship between log CA-125 and ovarian cancer diagnosis was non-linear, so restricted cubic splines were used. Models contained 3, 4 or 5 knots: the model that produced the smallest Akaike Information Criterion was taken forward [23]. Knots were subsequently placed equally at standard percentiles of the marginal distribution of the variable. The predicted incidences of ovarian cancer following a CA-125 result of 35U/ml, and the predicted CA-125 level required to reach the 3% ovarian cancer incidence rate (recommended by the NICE NG12 guideline [5]), were extracted.

As borderline ovarian tumours are included alongside invasive ovarian tumours in current NICE guidelines [4] but are less likely to raise serum CA-125 levels [24], a sensitivity analysis repeated the analyses using invasive ovarian cancer as the outcome, in accordance with previous research [6]. The ICD-O morphology and behaviour codes used to define invasive and borderline tumours can be found in Supplementary 1.

### Sample size calculations

A power calculation determined that a sample size of 1,118 was sufficient to detect a cancer incidence of 3% with a margin of error of < 1% point. Analyses were conducted using Stata MP version 18.0. Results were reported in accordance with The Reporting of Studies Collected Using Observational Routinely-Collected Heath Data (RECORD) statement [25], as an extension of Strengthening and Reporting of Observational Studies in Epidemiology (STROBE) statement [26] (Supplementary 2).

### Patient and public involvement

This study was developed in consultation with an existing Patient and Public Involvement and Engagement (PPIE) group, as part of a larger program of research. Four women were recruited especially for this study, comprising with at least one woman from each of the main ethnic groups analysed: White, Black, and Asian. Their thoughts and interpretation have helped shape the discussion.

## Results

### Patient characteristics

**Fig. 1:**
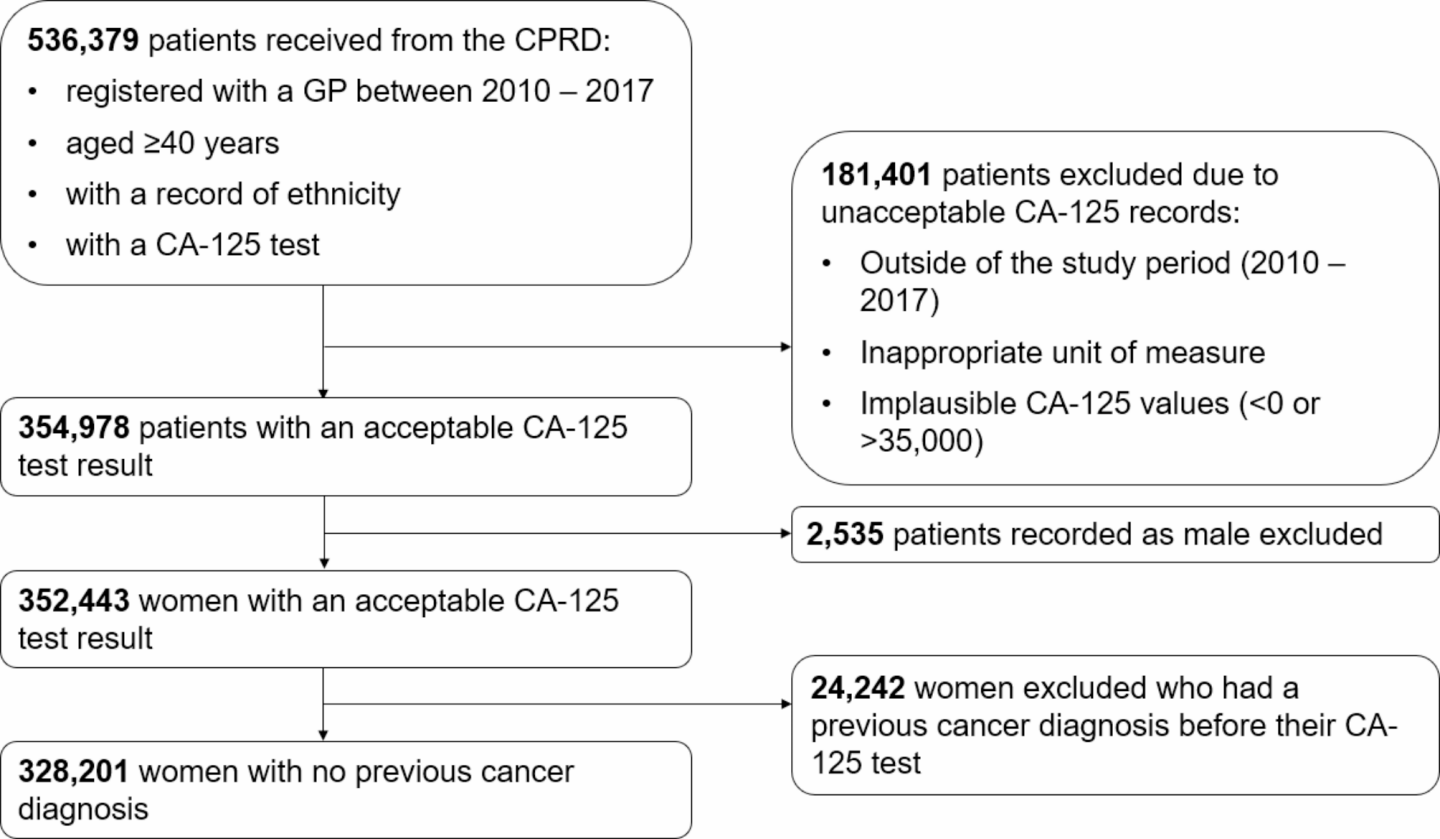
The cohort selection process

The cohort included 328,201 women aged ≥ 40 years with an acceptable CA-125 result in our study period with no prior cancer diagnosis (Fig. 1).

Patient characteristics are summarised in Table 1. A total of 328,201 women had a CA-125 test result in our study period (2010–2017). The majority were White (90%), followed by Asian (5%), and Black (3%). Women from the Other and Mixed ethnic groups each represented less than 1% of the study population, and there were 2,434 (< 1%) patients with record of unknown ethnicity who were included in the total figures. There were variations in patient characteristics across the ethnic groups. Most notably, White women were older (median 56 years vs. 50 years for both Asian and Black women) and Black women had higher deprivation scores (42% in the highest IMD quintile vs. 13% and 24% for White and Asian women, respectively). Median CA-125 levels were similar across the ethnic groups, although a lower proportion of White women had a high CA-125 result compared to Black and Asian women.

**Table 1:** Patient characteristics of the study cohort

Whole cohort							
	**White**	**Asian**	**Black**	**Other**	**Mixed**	**Total**	
**Patients** *n* (%)	294,816 (90%)	15,783 (5%)	9,473 (3%)	2,889 (< 1%)	2,806 (< 1%)	328,201 (100%)	
**Age** median (IQR)	56 (47–68)	50 (44–60)	50 (45–58)	50 (44–57)	49 (44–57)	55 (47–67)	
**Highest IMD quintile** (%)	13	24	42	25	23	15	
**Highest CMS tertile** (%)	23	21	19	14	14	8	
**CA-125 result** median (95% CI)	12 (8–18)	11 (8–17)	10 (7–16)	11 (7–17)	12 (8–18)	12 (8–18)	
**High CA-125** % (95% CI)	6.8 (6.7–6.9)	7.4 (7.0–7.9)	7.3 (6.8–7.9)	7.4 (6.5–8.4)	6.6 (5.7–7.5)	6.9 (6.8–7.0)	

### Ovarian cancer incidence

The one-year ovarian cancer incidence was 0.8% with 2,756 women in our cohort receiving an ovarian cancer diagnosis within one-year of their CA-125 test, including 2,589 White, 81 Asian, 43 Black, 10 Other, 10 Mixed, and 23 women with unknown ethnicity. There were too few ovarian cancer cases from the Mixed and Other groups to draw meaningful comparisons regarding ovarian cancer incidence. Consequently, these women were dropped from the analysis.

Table 2 outlines the one-year incidence of an ovarian cancer diagnosis and the incidence of an advanced stage diagnosis for White, Asian, and black women. White women were almost twice as likely to be diagnosed with ovarian cancer compared to Asian and black women (0.9% vs. 0.5% for both Asian and black women). Most women with ovarian cancer had an invasive epithelial ovarian cancer (≥ 95%) (supplementary 3). More black women were diagnosed with serous ovarian cancer than White or Asian women, and there were no black women in our cohort who were diagnosed with mucinous ovarian cancer (supplementary 3). Data on stage at diagnosis were available for 84% of patients. Black women were more likely to be diagnosed at an advanced stage compared to White and Asian women (67% vs. 58% for both White and Asian women). Median CA-125 levels in women with ovarian cancer were similar across the ethnic groups, however in women with advanced stage cancer they were higher for Asian women (751U/ml (95% CI 548 to 1,531U/ml)) compared to white women (496 U/ml (95% CI 462 to 531U/ml))

**Table 2:** Unadjusted one-year ovarian cancer incidence and advanced stage incidence

	White	Asian	Black	Total
**One-year ovarian cancer incidence** % (95% CI)	0.88 (0.84–0.91)	0.51 (0.40–0.63)	0.45 (0.33–0.61)	0.84 (0.81–0.87)
** Normal CA-125** % (95% CI)	0.19 (0.18–0.21)	0.16 (0.10–0.24)	0.04 (0.01–0.12)	0.19 (0.17–0.20)
** High CA-125** % (95% CI)	10.2 (9.8–10.6)	4.9 (3.8–6.3)	5.6 (4.0–7.6)	9.6 (9.3–10.1)
** Median CA-125 level** (95% CI)	192 (172–218)	217 (74.3–589)	256 (111–701)	193 (173–218)
Advanced stage* % (95% CI)*	57.6 (55.6–59.7)	58.3 (46.1–69.8)	66.7 (48.2–69.8)	57.6 (55.6–59.7)
** Normal CA-125** % (95% CI)	18.2 (14.4–21.8)	18.8 (4.0–45.6)	66.7 (9.4–99.2)	18.0 (14.6–21.8)
** High CA-125** % (95% CI)	67.8 (65.5–70.0)	69.6 (55.9–81.2)	66.7 (47.2–82.7)	67.7 (65.5–69.8)
** Median CA-125 level** (95% CI)	496 (462–531)	751 (548–1,531)	308 (65.2–1,512)	498 (462–531)

* the percentage of ovarian cancer patients who were diagnosed at an advanced stage

### Diagnostic performance of CA-125 for predicting ovarian cancer, by ethnicity

The diagnostic performance of CA-125 is outlined in Table 3 for White, Asian, and Black women and the results for Other and Mixed women is supplied in Supplementary 4. A high CA-125 result had the highest sensitivity in the detection of ovarian cancer for Black women: 90.7% (95% CI 90.1 to 91.2%) vs. 79.4% (95% CI 79.3 to 79.6%) for White women and 71.6% (95% CI 70.9 to 72.3%) for Asian women. The specificity was marginally higher for White women: 93.8% (95% CI 93.7 to 93.9%) vs. 92.9% (95% CI 92.5 to 93.3%) for Asian women and 93.1% (95% CI 92.5 to 93.6%) for Black women. The positive predictive values (PPVs) of a high CA-125 result were approximately twice as high for White women: 10.2% (95% CI 10.1 to 10.3%) compared 4.9% (95% CI 4.6 to 5.3%) for Asian and 5.6% (95% CI 5.2 to 6.1%) for Black women. The negative predictive values were similar across the ethnic groups. The AUC was extremely high for all ethnic groups (≥ 0.90), but highest for Black women (0.96, 95% CI 0.92 to 0.99).

The diagnostic performance was largely unaffected following the exclusion of borderline tumours and women aged 50 years (Supplementary 4). In contrast to the unadjusted differences in PPVs of a high CA-125 result by ethnicity, adjustments for age group in 10-year categories, CMS, IMD, and year of test estimated the PPVs to be similar across all ethnic groups: 9.31% (95% CI 8.83 to 9.82) for White women, 7.84 (95% CI 6.06 to 10.1) for Asian women, and 8.39 (95% CI 6.12 to 11.4) for Black women. This reduction in differences was largely due to the adjustment for age-group (Supplementary 5).

**Table 3:** The diagnostic accuracy of CA-125, by ethnicity. Sensitivity, specificity, PPV, NPV, and the diagnostic OR used the CA-125 threshold of ≥ 35/ml. The diagnostic odds ratio reported the odds of being diagnosed with ovarian cancer following a high CA-125 result, relative to a normal CA-125 result. adjustments made for age group in 10-year categories, CMS, IMD, and year of test

Diagnostic accuracy of CA-125 for the detection of ovarian cancer				
**%** (95% CI)	**White**	**Asian**	**Black**	**All**
**Sensitivity**	79.4 (79.3–79.6)	71.6 (70.9–72.3)	90.7 (90.1–91.2)	79.5 (79.3–79.6)
**Specificity**	93.8 (93.7–93.9)	92.9 (92.5–93.3)	93.1 (92.5–93.6)	93.7 (93.7–93.8)
**PPV**	10.2 (10.1–10.3)	4.9 (4.6–5.3)	5.6 (5.2–6.1)	9.7 (9.6–9.8)
**Adjusted PPV**	9.3 (8.8–9.8)	7.8 (6.1–10.1)	8.4 (6.1–11.4)	9.1 (8.7–9.6)
**NPV**	99.8 (99.8–99.8)	99.9 (99.8–99.9)	99.95 (99.9–100)	99.8 (99.8–99.8)
**AUC**	0.93 (0.92–0.93)	0.90 (0.86–0.94)	0.96 (0.92–0.99)	0.93 (0.92–0.93)
**Adjusted AUC**	0.90 (0.90–0.91)	0.86 (0.81–0.91)	0.95 (0.91–0.99)	0.90 (0.90–0.91)
**Adjusted diagnostic OR**	70.7 (64.0–78.2)	55.8 (33.8–92.2)	187.4 (66.4–528.4)	71.3 (64.7–78.6)

The degree of association of an abnormal CA-125 result with incident ovarian cancer, relative to a normal result, differed significantly by ethnicity (interaction *p-*value = 0.02). However, adjustment for all covariates (age group in 10-year categories, CMS, IMD, and year of test), and for age group alone, nullified the interaction. The diagnostic odds ratio was over three times higher for Black women compared to White or Asian women: 187.4 (95% CI 66.4 to 528.4) for Black women, 70.7 (95% CI 64.0 to 78.2) for White women, and 55.8 (95% CI 33.8 to 92.2) for Asian women, although the confidence intervals for Black women were extremely wide so this was not a significant difference (Table 3).

The adjusted incidences and odds ratios of an incident ovarian cancer diagnosis for all ethnic groups can be found in Supplementary 6a. The results were largely unaffected when invasive ovarian cancer formed the outcome (Supplementary 6b).

### Association of a high CA-125 result with the diagnosis of advanced stage ovarian cancer by ethnicity

For women who received an ovarian cancer diagnosis, the adjusted PPVs of an advanced stage diagnosis by CA-125 result were calculated. Adjustments were made for age group in 10-year categories, ovarian cancer histology, CMS, IMD, and year of test. The adjusted PPVs of an advanced stage diagnosis following a raised CA-125 result were similar for all women: 75.9% for White women (95% CI 72.6 to 78.9%), 69.6% for Asian women (95% CI 72.3 to 93.7), and 66.8% (95% CI 41.4 to 85.1) for Black women.

The point estimate of the odds ratio of being diagnosed at an advanced stage, relative to an early stage, following a raised CA-125 result in an ovarian cancer population was higher for Asian women (OR 15.7 (95% CI 2.66 to 93.1) vs. 6.08 (95% CI 4.13 to 8.96%) for White women and 0.40 (95% CI 0.02 to 9.25%) for Black women), although this was not significant. The degree of association between an elevated CA-125 result and being diagnosed with an advanced stage of ovarian cancer, relative to CA-125 result, did not differ by ethnicity.

A table of these results, including for the Other and Mixed ethnic groups can be found in Supplementary table 6c.

### The estimated incidence of ovarian cancer by CA-125 level, by ethnicity

**Fig. 2:**
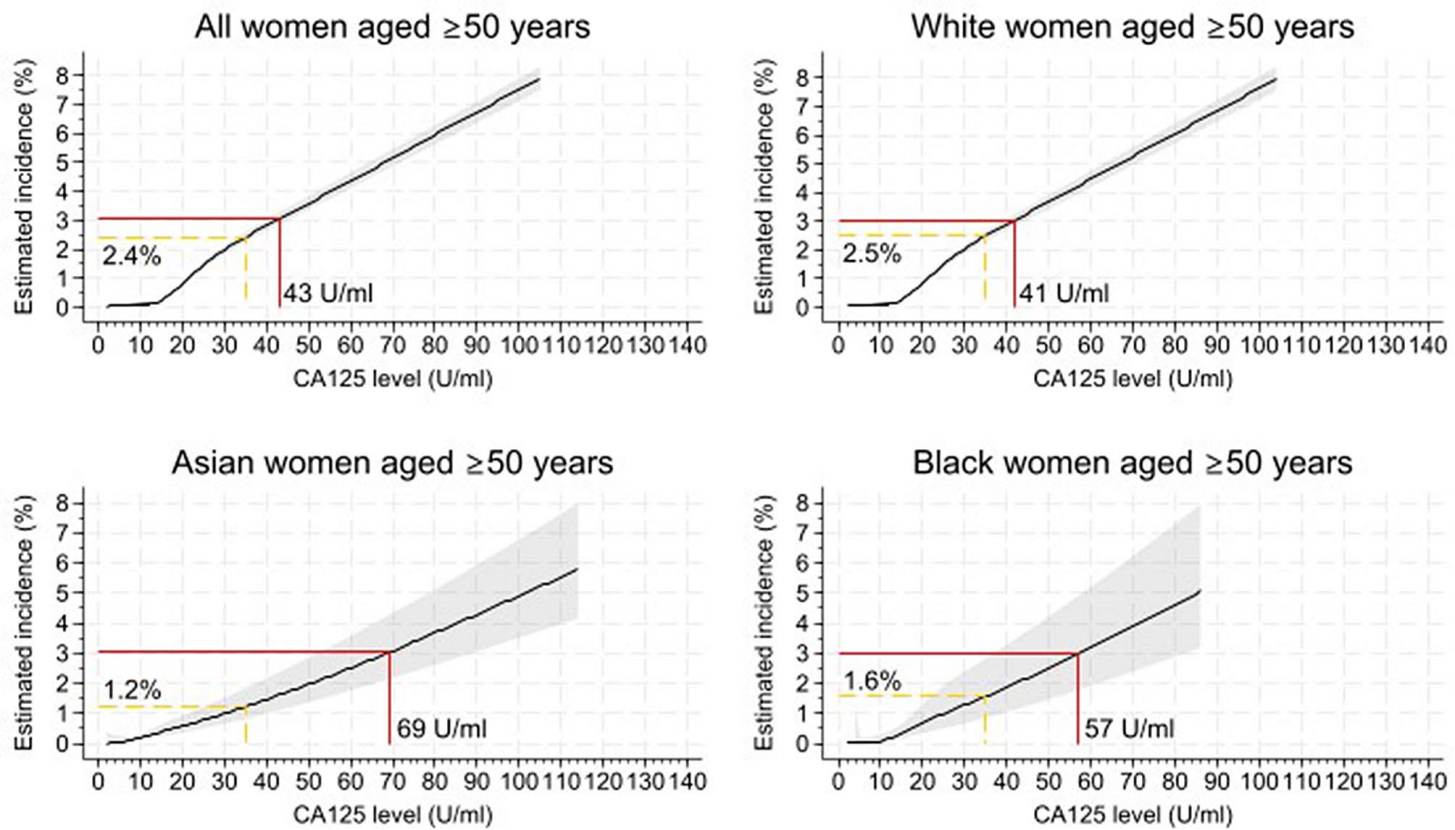
The predicted incidence of ovarian cancer by CA-125 level and ethnicity for women aged ≥ 50 years up to 8%. The red line indicates the estimated CA-125 level to represent a 3% incidence of ovarian cancer and the dashed yellow line predicts the incidence of ovarian cancer at a CA-125 level of 35U/ml. The shading represents the 95% CIs.

The predicted one-year incidence of ovarian cancer by CA-125 level and ethnicity for women aged ≥ 50 years is shown in Fig. 2. The overall estimated incidence of ovarian cancer at a CA-125 level of 35U/ml was 2.4% (95% CI 2.3 to 2.6%) and the estimated CA-125 level to correspond to a 3% incidence of ovarian cancer was 43 U/ml (95% CI 40 to 45 U/ml). These figures were similar for White women: 2.5% (95% CI 2.3 to 2.7%) and 41 U/ml (95% CI 39 to 44 U/ml), respectively. The predicted incidence for Asian women following a CA-125 level of 35U/ml was half the predicted incidence for White women (1.2%, 95% CI 0.8 to 1.8%). Similarly, Asian women reached the estimated 3% ovarian cancer incidence rate at higher CA-125 levels (69 U/ml (95% CI 52 to 89 U/ml)). For Black women, a CA-125 level of 35U/ml represented a lower estimated incidence of ovarian cancer (1.6%, 95% CI 0.9 to 2.8%) than White women, and a lower CA-125 level of 57 U/ml (95% CI 37 to 82 U/ml) to correspond to a predicted incidence rate of 3% of ovarian cancer. However, due to an insufficient number of Black women diagnosed with ovarian cancer aged  ≥ 50 years, the confidence intervals were too wide to confirm differences.

Similar values were observed for the prediction of invasive ovarian cancers in women aged ≥ 50 years (Supplementary 7). The predicted incidence of ovarian cancer by CA-125 level and ethnicity for all women (including women aged ≥ 40 and the Other and Mixed ethnicities) can also be found in Supplementary 7.

Application of the ethnicity-specific thresholds resulted in increased specificity and PPVs for all ethnic groups, at the expense of sensitivity, particularly for Asian and Black women who would see a much larger reduction in sensitivity, and increase in false negatives, than White women. The diagnostic performance of the ethnicity-specific thresholds modelled in Fig. 2 is outlined in Supplementary 8a, along with a 2 × 2 table comparison of the ethnicity-specific thresholds and the current 35U/mL thresholds in Supplementary 8b.

## Discussion

### Summary of findings

This is the largest study of the diagnostic performance of CA-125 in predicting ovarian cancer in women in English primary care and the first to consider the diagnostic performance of CA-125 in women of different ethnic groups. As expected, ovarian cancer incidence in women who had a CA-125 test was highest in White women, although this was partly due to differences in the distribution of age, deprivation status, and presence and/or severity of comorbidities across the different ethnic groups. CA-125 was more sensitive with a higher AUC for Black women and was least sensitive for Asian women. White women experienced higher PPVs which likely reflected the increased incidence in these women. There was a significant interaction effect between ethnicity and CA-125 result with incident ovarian cancer diagnosis, but the nullification of this effect by adjusting for covariates suggested that the effect was caused by age, comorbidities, and deprivation scores, rather than ethnicity.

Similar to existing reports [27], Black women were more likely to be diagnosed at an advanced stage, which may be partly attributed to sociodemographic factors and tumour histology. Finally, our work has shown that the CA-125 level corresponding to an estimated 3% ovarian cancer incidence threshold was highest for Asian women, with White women estimated to reach the 3% threshold at much lower values.

### Strengths and limitations

This was a large cohort study using primary care data from the CPRD which includes approximately 20% of the consulting primary care population in the UK [13] with a suitable representation of ethnicity [28]. Our study included 325,767 eligible women with a CA-125 result and an ethnic group assignment. We captured important data on covariates that were likely to influence CA-125 levels including age, CMS, IMD, year of test, ovarian cancer histology, and whether the ovarian cancer was borderline or invasive and accounted for these throughout our analyses.

Despite the large size of our cohort, there were an inadequate number of ovarian cancers in the Other and Mixed ethnic groups for meaningful comparisons for these groups. Previous research also struggled to draw any conclusions about these groups due to their small sample sizes and heterogeneity within groups [18]. In addition, we did not account for inter-ethnic differences, such as the differences between the East and South Asian ethnic groups which were highlighted during the COVID-19 pandemic [29].

While the patient selection criteria ensured patients who had previously been diagnosed with cancer were not included, and adjustments for CMS controlled for previously diagnosed conditions, it was not possible to account for undiagnosed conditions that may have been causing elevations of CA-125. Other conditions that are known to raise CA-125 include other types of cancer [6], benign pelvic tumours, ovarian hyperstimulation syndrome and other benign conditions [30]. These conditions may also be impacted by ethnicity.

As a consequence of using routinely collected healthcare record data in this study, we were unable to ascertain whether women from different ethnic groups present and report to primary care equally, or whether they were offered, or accepted, CA-125 tests at equal rates. People from some ethnic groups may present to primary care later than others due to factors including stigma [31], fear and fatalism [32], religion [33], differences in symptom awareness and additional barriers to healthcare [34]. As a crude measure, a comparison to ethnicity distribution in the England and Wales 2011 [35] and 2021 [36] census and the ethnicity distribution in CPRD Aurum and HES in 2011 [37] and 2021 [28] showed the ethnic distribution was similar to our cohort population, albeit with a slight underrepresentation of the Asian ethnic group.

### Comparison to existing literature

Funston, et al. (2020) [6] were the first to analyse the diagnostic performance of CA-125 in detecting ovarian cancer in primary care, using CPRD GOLD data. Their reported sensitivity, specificity, PPV, NPV and AUC aligns closely with our study, supporting the value of CA-125 as a diagnostic tool for ovarian cancer. A follow-up modelling study by the same authors [8] retained ethnicity (White vs. Non-White) as a significant covariate when predicting ovarian cancer using CA-125, although this model had similar performance metrics to one based solely on CA-125 level and age. Our study confirms that CA-125 performs well for women of all ethnicities but has higher sensitivity and discriminative power for Black women, and lower sensitivity for Asian women.

A recent study comparing the diagnostic performance of prostate-specific antigen levels for the detection of prostate cancer found estimated cancer incidence to be higher for Black men and lower for Asian men, compared to White men when adjusting for IMD, morbidity and year of test [18]. When using advanced stage disease as a proxy for clinically significant prostate cancers, this difference was eliminated between Black and White men, with incidence remaining lower for Asian men. As Black men without prostate cancer were found to have higher levels of PSA than White men [38], these results suggest that there may be increased levels of over-diagnosis of prostate cancer in Black men. This is not a conclusion reached by the present study in relation to CA-125 and ovarian cancer in Black women, highlighting the importance of assessing the performance of all diagnostic tests in patients of different ethnicities.

### Clinical implications

The increased sensitivity of CA-125 in Black women may be attributable to a higher proportion of Black women being diagnosed at an advanced stage compared to the other ethnic groups. It may also be reflective of the histology or the aggressiveness of the tumour that these women are more likely to be diagnosed with. Previous studies have indicated that serous and endometrioid ovarian cancers elevate CA-125 levels more than the other histological subtypes, and mucinous tumours do not increase CA-125 levels to the same extent [9, 39, 40]. Serous and endometrioid ovarian cancers were most common in Black women in our cohort and there were no Black women diagnosed with mucinous ovarian cancer (Supplementary 3). This could explain the much more equal distribution of predicted stage at diagnosis across the ethnic groups after adjusting for covariates, including ovarian cancer histology.

Our study may suggest that Asian women could benefit from an increased CA-125 threshold to initiating further ovarian cancer investigations. This adjustment would reduce the likelihood of unnecessary investigations in these women, by equalising the probability of triggering ovarian cancer investigations across different ethnic groups. However, application of the ethnicity-specific thresholds modelled in Fig. 2 to equalise the incidence of ovarian cancer represented by the threshold would result in increased specificity and PPVs for all ethnic groups, at the expense of sensitivity, particularly for Asian and Black women who would see a much larger reduction in sensitivity than White women. This could lead to diagnosis delays to proportionately more Asian and Black women. Additionally, applying a biological reference range on the basis of a social construct such as ethnicity may perpetuate health inequalities [41, 42]. Incidentally, in 2021 a joint statement from the National Kidney Foundation and the American Society of Nephrology advocated for a race-free calculation to replace the race-specific calculation to estimate glomerular filtration rate to detect and manage kidney disease [43]. An alternative approach would be to use age-specific thresholds as suggested in previous work [6], which would have different impacts across ethnic groups addressing some of these differences without making judgements based on ethnicity. This is supported by the observation that adjustment for age-group eliminated ethnic variations in the PPVs for ovarian cancer diagnosis following a raised CA-125 result, as well as the interaction effect between CA-125 result and ethnicity on incident ovarian cancer diagnosis.

## Conclusion

Clinicians should continue to offer CA-125 tests to all women who present to primary care with non-specific symptoms of ovarian cancer, and to refer women for further ovarian cancer investigations equally, regardless of ethnicity. Future research should consider whether other diagnostic tests perform equally well for all ethnic groups and should also consider the socio-economic challenges that some patients may face when accessing healthcare.

### Electronic supplementary material

Below is the link to the electronic supplementary material.

Supplementary Material 1

Supplementary Material 2

Supplementary Material 3

Supplementary Material 4

Supplementary Material 5

Supplementary Material 6

Supplementary Material 7

Supplementary Material 8

## Data Availability

Data were obtained from the CPRD with linkage to HES and NCRAS and were under license for this study. Therefore, restrictions may apply to the availability of these data.

## References

[1] Cancer Research UK. Ovarian cancer statistics. 2022.

[2] National Health Service. Online version of the NHS Long Term Plan [Internet]. [cited 2023 Nov 20]. https://www.longtermplan.nhs.uk/

[3] National Health Service. Implementing a timed gynaecology cancer diagnostic pathway. 2023.

[4] National Institute of Health Excellence (NICE). Ovarian cancer: recognition and initial management CG122. 2011.31815390

[5] National Institute of Health and Care Excellence. Suspected cancer: recognition and referral. 2015. 26180880

[6] Funston G, Hamilton W, Abel G, Crosbie EJ, Rous B, Walter FM. The diagnostic performance of CA125 for the detection of ovarian and non-ovarian cancer in primary care: a population-based cohort study. PLoS Med. 2020;17(10):e1003295.33112854 10.1371/journal.pmed.1003295PMC7592785

[7] Barr CE, Funston G, Jeevan D, Sundar S, Mounce LTA, Crosbie EJ. The performance of HE4 alone and in combination with CA125 for the detection of Ovarian Cancer in an Enriched Primary Care Population. Cancers (Basel). 2022;14(9):2124.35565253 10.3390/cancers14092124PMC9101616

[8] Funston G, Abel G, Crosbie EJ, Hamilton W, Walter FM. Could Ovarian Cancer Prediction models improve the triage of symptomatic women in primary care? A modelling study using routinely Collected Data. Cancers (Basel). 2021;13(12):2886.34207611 10.3390/cancers13122886PMC8228892

[9] Babic A, Cramer DW, Kelemen LE, Köbel M, Steed H, Webb PM, et al. Predictors of pretreatment CA125 at ovarian cancer diagnosis: a pooled analysis in the Ovarian Cancer Association Consortium. Cancer Causes Control. 2017;28(5):459–68.28050675 10.1007/s10552-016-0841-3PMC5593071

[10] Pauler DK, Menon U, McIntosh M, Symecko HL, Skates SJ, Jacobs IJ. Factors influencing serum CA125II levels in healthy postmenopausal women. Cancer Epidemiol Biomarkers Prev. 2001;10(5):489–93. 11352859

[11] Johnson CC, Kessel B, Riley TL, Ragard LR, Williams CR, Xu JL, et al. The epidemiology of CA-125 in women without evidence of ovarian cancer in the prostate, lung, colorectal and ovarian Cancer (PLCO) screening trial. Gynecol Oncol. 2008;110(3):383–9.18586313 10.1016/j.ygyno.2008.05.006PMC3744195

[12] Wolf A, Dedman D, Campbell J, Booth H, Lunn D, Chapman J, et al. Data resource profile: Clinical Practice Research Datalink (CPRD) Aurum. Int J Epidemiol. 2019;48(6):1740–g1740.30859197 10.1093/ije/dyz034PMC6929522

[13] Clinical Practice Research Database. Release Notes: CPRD Aurum May 2022. 2022.

[14] Herbert A, Wijlaars L, Zylbersztejn A, Cromwell D, Hardelid P. Data Resource Profile: Hospital Episode Statistics Admitted Patient Care (HES APC). Int J Epidemiol [Internet]. 2017 Aug 1 [cited 2021 Sep 13];46(4):1093–1093i. https://academic.oup.com/ije/article/46/4/1093/3072145 10.1093/ije/dyx015 PMC5837677 28338941

[15] Martins T, Abel G, Ukoumunne OC, Mounce LTA, Price S, Lyratzopoulos G et al. Ethnic inequalities in routes to diagnosis of cancer: a population-based UK cohort study. Br J Cancer [Internet]. 2022 Jun 6 [cited 2022 Aug 19]; https://pubmed.ncbi.nlm.nih.gov/35661833/10.1038/s41416-022-01847-x PMC9427836 35661833

[16] Mathur R, Bhaskaran K, Chaturvedi N, Leon DA, van Staa T, Grundy E, et al. Completeness and usability of ethnicity data in UK-based primary care and hospital databases. J Public Health (United Kingdom). 2014;36(4):684–92. 10.1093/pubmed/fdt116 PMC4245896 24323951

[17] Mathur R, Palla L, Farmer RE, Chaturvedi N, Smeeth L. Ethnic differences in the severity and clinical management of type 2 diabetes at time of diagnosis: a cohort study in the UK Clinical Practice Research Datalink. Diabetes Res Clin Pract. 2020;160:108006.31923438 10.1016/j.diabres.2020.108006PMC7042884

[18] Down L, Barlow M, Bailey SER, Mounce LTA, Merriel SWD, Watson J, et al. Association between patient ethnicity and prostate cancer diagnosis following a prostate-specific antigen test: a cohort study of 730,000 men in primary care in the UK. BMC Med. 2024;22(1):82.38424555 10.1186/s12916-024-03283-5PMC10905783

[19] Henson KE, Elliss-Brookes L, Coupland VH, Payne E, Vernon S, Rous B et al. Data Resource Profile: National Cancer Registration Dataset in England. Int J Epidemiol [Internet]. 2020 Feb 1 [cited 2021 Sep 13];49(1):16–16h. https://academic.oup.com/ije/article/49/1/16/5476570 10.1093/ije/dyz076 PMC7124503 31120104

[20] Department for Communities and Local Government. The English Index of Multiple Deprivation (IMD) 2015-Guidance [Internet]. 2015 [cited 2022 Dec 2]. https://www.gov.uk/government/statistics/english-indices-of-deprivation-2015

[21] Payne RA, Mendonca SC, Elliott MN, Saunders CL, Edwards DA, Marshall M et al. Development and validation of the Cambridge Multimorbidity Score. CMAJ [Internet]. 2020 Feb 3 [cited 2022 Nov 2];192(5):E107–14. https://pubmed.ncbi.nlm.nih.gov/32015079/10.1503/cmaj.190757 PMC7004217 32015079

[22] Skates SJ, Mai P, Horick NK, Piedmonte M, Drescher CW, Isaacs C, et al. Large prospective study of ovarian cancer screening in high-risk women: CA125 cut-point defined by menopausal status. Cancer Prev Res (Phila). 2011;4(9):1401–8.21893500 10.1158/1940-6207.CAPR-10-0402PMC3172691

[23] Harrell F. Regression modeling strategies. New York: Springer; 2001.

[24] Fischerova D, Zikan M, Dundr P, Cibula D. Diagnosis, treatment, and Follow-Up of Borderline ovarian tumors. Oncologist. 2012;17(12):1515–33.23024155 10.1634/theoncologist.2012-0139PMC3528384

[25] Benchimol EI, Smeeth L, Guttmann A, Harron K, Moher D, Petersen I, et al. The REporting of studies conducted using Observational routinely-collected health data (RECORD) Statement. PLoS Med. 2015;12(10):e1001885.26440803 10.1371/journal.pmed.1001885PMC4595218

[26] von Elm E, Altman DG, Egger M, Pocock SJ, Gøtzsche PC, Vandenbroucke JP. The Strengthening the Reporting of Observational Studies in Epidemiology (STROBE) statement: guidelines for reporting observational studies. Lancet [Internet]. 2007 [cited 2022 Sep 8];370(9596):1453–7. https://pubmed.ncbi.nlm.nih.gov/18064739/10.1016/S0140-6736(07)61602-X 18064739

[27] Fry A, White B, Nagarwalla D, Shelton J, Jack RH. Relationship between ethnicity and stage at diagnosis in England: a national analysis of six cancer sites. BMJ Open. 2023;13(1):e062079.36702581 10.1136/bmjopen-2022-062079PMC9884890

[28] Shiekh SI, Harley M, Ghosh RE, Ashworth M, Myles P, Booth HP, et al. Completeness, agreement, and representativeness of ethnicity recording in the United Kingdom’s Clinical Practice Research Datalink (CPRD) and linked Hospital Episode statistics (HES). Popul Health Metr. 2023;21(1):3.36918866 10.1186/s12963-023-00302-0PMC10013294

[29] Mathur R, Rentsch CT, Morton CE, Hulme WJ, Schultze A, MacKenna B, et al. Ethnic differences in SARS-CoV-2 infection and COVID-19-related hospitalisation, intensive care unit admission, and death in 17 million adults in England: an observational cohort study using the OpenSAFELY platform. Lancet. 2021;397(10286):1711–24.33939953 10.1016/S0140-6736(21)00634-6PMC8087292

[30] Daoud E, Bodor G. CA-125 concentrations in malignant and nonmalignant disease. Clin Chem. 1991;37(11):1968–74.1934471 10.1093/clinchem/37.11.1968

[31] Vrinten C, Gallagher A, Waller J, Marlow LAV. Cancer stigma and cancer screening attendance: a population based survey in England. BMC Cancer. 2019;19(1):566.31185949 10.1186/s12885-019-5787-xPMC6561035

[32] Vrinten C, Wardle J, Marlow LA. Cancer fear and fatalism among ethnic minority women in the United Kingdom. Br J Cancer. 2016;114(5):597–604.26867159 10.1038/bjc.2016.15PMC4782206

[33] Afsah YR, Kaneko N. Barriers to cervical cancer screening faced by immigrant muslim women: a systematic scoping review. BMC Public Health. 2023;23(1):2375.38037019 10.1186/s12889-023-17309-9PMC10687813

[34] Niksic M, Rachet B, Warburton FG, Forbes LJL. Ethnic differences in cancer symptom awareness and barriers to seeking medical help in England. Br J Cancer. 2016;115(1):136–44.27280638 10.1038/bjc.2016.158PMC4931374

[35] Office for National Statistics. Ethnicity and National Identity in England and Wales: 2011. 2011.

[36] Office for National Statistics. Ethnic group, England and Wales: Census 2021. 2021.

[37] Mathur R, Bhaskaran K, Chaturvedi N, Leon DA, vanStaa T, Grundy E, et al. Completeness and usability of ethnicity data in UK-based primary care and hospital databases. J Public Health (Bangkok). 2014;36(4):684–92. 10.1093/pubmed/fdt116 PMC4245896 24323951

[38] Barlow M, Down L, Mounce LTA, Merriel SWD, Watson J, Martins T, et al. Ethnic differences in prostate-specific antigen levels in men without prostate cancer: a systematic review. Prostate Cancer Prostatic Dis. 2022;26(2):249–56.36456698 10.1038/s41391-022-00613-7PMC10247367

[39] Duffy MJ, Bonfrer JM, Kulpa J, Rustin GJS, Soletormos G, Torre GC, et al. CA125 in ovarian cancer: European Group on Tumor markers guidelines for clinical use. Int J Gynecol Cancer. 2005;15(5):679–91.16174214 10.1136/ijgc-00009577-200509000-00001

[40] Sölétormos G, Duffy MJ, Othman Abu Hassan S, Verheijen RHM, Tholander B, Bast RC, et al. Clinical use of Cancer biomarkers in epithelial ovarian Cancer: updated guidelines from the European Group on Tumor markers. Int J Gynecologic Cancer. 2016;26(1):43–51. 10.1097/IGC.0000000000000586 PMC4679342 26588231

[41] Weyand AC, McGann PT. Eliminating race-based reference ranges in haematology: a call to action. Lancet Haematol. 2021;8(6):e462–6.34048684 10.1016/S2352-3026(21)00106-X

[42] Cerdeña JP, Plaisime MV, Tsai J. From race-based to race-conscious medicine: how anti-racist uprisings call us to act. Lancet. 2020;396(10257):1125–8.33038972 10.1016/S0140-6736(20)32076-6PMC7544456

[43] Delgado C, Baweja M, Crews DC, Eneanya ND, Gadegbeku CA, Inker LA, et al. A Unifying Approach for GFR Estimation: recommendations of the NKF-ASN Task Force on reassessing the inclusion of race in diagnosing kidney disease. Am J Kidney Dis. 2022;79(2):268–e2881.34563581 10.1053/j.ajkd.2021.08.003

