# Supplementary material for: The diagnostic performance of CA-125 for the detection of ovarian cancer in women from different ethnic groups: a cohort study of English primary care data
Source: J Ovarian Res. 2024 Aug 26;17:173. doi: 10.1186/s13048-024-01490-5 (PMC11346194; doi:10.1186/s13048-024-01490-5)
Supplement: Supplementary file 1 — Supplementary Material 1 [file 13048_2024_1490_MOESM1_ESM.docx]

**Supplementary 1: categorisation of ICD-O morphology and behaviour codes.**

0 = no, 1= yes

| **ICD-O morphology and behaviours** | **Morphology** | **Histology** | **Invasive** | **Borderline** |
| --- | --- | --- | --- | --- |
| 8000/1 | Neoplasm, uncertain whether benign or malignant | Unknown | 0 | 1 |
| 8000/3 | Neoplasm, malignant | Unknown | 1 | 0 |
| 8010/1 | Epithelial tumour, uncertain behaviour | Epithelial (other) | 0 | 1 |
| 8010/3 | Carcinoma NOS | Epithelial (unknown) | 1 | 0 |
| 8020/3 | Carcinoma, undifferentiated NOS | Epithelial (other) | 1 | 0 |
| 8041/3 | Small cell carcinoma NOS | Epithelial (other) | 1 | 0 |
| 8070/3 | Squamous cell carcinoma NOS | Epithelial (other) | 1 | 0 |
| 8140/3 | Adenocarcinoma NOS | Epithelial (unknown) | 1 | 0 |
| 8240/3 | Carcinoid tumor NOS | Non-epithelial | 1 | 0 |
| 8246/3 | Neuroendocrine carcinoma | Epithelial (other) | 1 | 0 |
| 8310/3 | Clear cell adenocarcinoma NOS | Clear cell | 1 | 0 |
| 8313/1 | Clear cell adenofibroma of borderline malignancy | Clear cell | 0 | 1 |
| 8323/3 | Mixed cell adenocarcinoma | Epithelial (other) | 1 | 0 |
| 8380/3 | Endometrioid carcinoma | Endometrioid | 1 | 0 |
| 8440/3 | Cystadenocarcinoma NOS | Epithelial (unknown) | 1 | 0 |
| 8441/1 | Serous cystadenoma, borderline malignancy | Serous | 0 | 1 |
| 8441/3 | Serous cystadenocarcinoma NOS | Serous | 1 | 0 |
| 8442/3 | Serous cystadenoma, borderline malignancy | Serous | 0 | 1 |
| 8442/5 | Serous cystadenoma, microinvasion | Serous | 0 | 1 |
| 8460/3 | Papillary serous cystadenocarcinoma | Serous | 1 | 0 |
| 8461/3 | Serous surface papillary carcinoma | Serous | 1 | 0 |
| 8462/3 | Papillary serous cystadenoma, borderline malignancy | Serous | 0 | 1 |
| 8462/5 | Papillary serous cystadenoa, microinvasion | Serous | 0 | 1 |
| 8470/3 | Mucinous cystadenocarcinoma NOS | Mucinous | 1 | 0 |
| 8472/3 | Mucinous cystadenoma, borderline malignancy | Mucinous | 0 | 1 |
| 8472/5 | Mucinous cystadenoma, microinvasive | Mucinous | 0 | 1 |
| 8473/3 | Papillary mucinous cystadenoma, borderline malignancy | Mucinous | 0 | 1 |
| 8480/3 | Mucinous adenocarcinoma | Mucinous | 1 | 0 |
| 8620/1 | Granulosa cell tumor NOS | Non-epithelial | 1 | 0 |
| 8620/3 | Granulosa cell tumor, malignant | Non-epithelial | 1 | 0 |
| 8631/3 | Sertoli-Leydig cell tumour, poorly differentiated | Non-epithelial | 1 | 0 |
| 8890/3 | Leiomyosarcoma NOS | Non-epithelial | 1 | 0 |
| 8950/3 | Mullerian mixed tumor | Epithelial (other) | 1 | 0 |
| 8980/3 | Carcinosarcoma, NOS | Epithelial (other) | 1 | 0 |
| 9014/1 | Serous adenofibroma of borderline malignancy | Serous | 0 | 1 |
| 9014/3 | Serous adenocarcinofibroma | Serous | 1 | 0 |
| 9015/1 | Mucinous adenofibroma of borderline malignancy | Mucinous | 0 | 1 |
| 9071/3 | Endodermal sinus tumour | Non-epithelial | 1 | 0 |
| 9080/3 | Teratoma, malignant NOS | Non-epithelial | 1 | 0 |
| 9085/3 | Mixed germ cell tumor | Non-epithelial | 1 | 0 |
