# Supplementary material for: The diagnostic performance of CA-125 for the detection of ovarian cancer in women from different ethnic groups: a cohort study of English primary care data
Source: J Ovarian Res. 2024 Aug 26;17:173. doi: 10.1186/s13048-024-01490-5 (PMC11346194; doi:10.1186/s13048-024-01490-5)
Supplement: Supplementary file 3 — Supplementary Material 3 [file 13048_2024_1490_MOESM3_ESM.docx]

**Supplementary 3: ovarian cancer histology by ethnic group**

|  |  | **White** | **Asian** | **Black** | **Other** | **Mixed** | **Unknown** | **Total** |
| --- | --- | --- | --- | --- | --- | --- | --- | --- |
|  |  |  |  |  |  |  |  |  |
| **Serous** | n | 1,094 | 39 | 24 | 3 | 2 | 7 | 1,169 |
|  | % | 53.7 | 55.7 | 64.9 | 50.0 | 28.6 | 46.7 | 53.8 |
|  |  |  |  |  |  |  |  |  |
| **Mucinous** | n | 164 | 7 | 0 | 0 | 1 | 0 | 172 |
|  | % | 8.1 | 10.0 | 0.0 | 0.0 | 14.3 | 0.0 | 7.9 |
|  |  |  |  |  |  |  |  |  |
| **Endometrioid** | n | 165 | 5 | 4 | 0 | 1 | 1 | 176 |
|  | % | 8.1 | 7.1 | 10.8 | 0.0 | 14.3 | 6.7 | 8.1 |
|  |  |  |  |  |  |  |  |  |
| **Clear cell** | n | 130 | 6 | 1 | 0 | 1 | 0 | 138 |
|  | % | 6.4 | 8.6 | 2.7 | 0.0 | 14.3 | 0.0 | 6.4 |
|  |  |  |  |  |  |  |  |  |
| **Epithelial - other** | n | 90 | 3 | 2 | 0 | 0 | 2 | 97 |
|  | % | 4.4 | 4.3 | 5.4 | 0.0 | 0.0 | 13.3 | 4.5 |
|  |  |  |  |  |  |  |  |  |
| **Epithelial - unknown** | n | 355 | 6 | 6 | 3 | 1 | 4 | 375 |
|  | % | 17.4 | 8.6 | 16.2 | 50.0 | 14.3 | 26.7 | 17.3 |
|  |  |  |  |  |  |  |  |  |
| **Non-epithelial** | n | 39 | 4 | 0 | 0 | 1 | 1 | 45 |
|  | % | 1.9 | 5.7 | 0.0 | 0.0 | 14.3 | 6.7 | 2.1 |
|  |  |  |  |  |  |  |  |  |
| **Total** | n | 2,037 | 70 | 37 | 6 | 7 | 15 | 2,172 |
|  | % | 100.0 | 100.0 | 100.0 | 100.0 | 100.0 | 100.0 | 100.0 |
