# Supplementary material for: The diagnostic performance of CA-125 for the detection of ovarian cancer in women from different ethnic groups: a cohort study of English primary care data
Source: J Ovarian Res. 2024 Aug 26;17:173. doi: 10.1186/s13048-024-01490-5 (PMC11346194; doi:10.1186/s13048-024-01490-5)
Supplement: Supplementary file 4 — Supplementary Material 4 [file 13048_2024_1490_MOESM4_ESM.docx]

**Supplementary 4. The diagnostic performance of CA-125 (35U/ml threshold) in detecting ovarian cancer, stratified by invasive cancer and women aged 50 years or over.**

|  |  | **Sensitivity** | **Specificity** | **PPV** | **NPV** | **AUC** |
| --- | --- | --- | --- | --- | --- | --- |
| **White** | All | 79.4  (79.3 – 79.6) | 93.8  (93.7 – 93.9) | 10.2  (10.1 – 10.3) | 99.8  (99.8 – 99.8) | 0.93  (0.92 – 0.93) |
|  | Invasive | 85.6  (85.5 – 85.7) | 93.8  (93.7 – 93.9) | 9.0  (8.9 – 9.1) | 99.8  (99.8 – 99.9) | 0.95  (0.94 – 0.95) |
|  | ≥50 years | 81.3  (81.2 – 81.5) | 94.5  (94.4 – 94.6) | 14.5  (14.4 – 14.7) | 99.8  (99.8 – 99.8) | 0.94  (0.93 – 0.94) |
| **Asian** | All | 71.6  (70.9 – 72.3) | 92.9  (92.5 – 93.3) | 4.9  (4.6 – 5.3) | 99.9  (99.8 – 99.9) | 0.90  (0.86 – 0.94) |
|  | Invasive | 75.4  (74.7 – 76.0) | 92.9  (92.4 – 93.2) | 4.4  (4.1 – 4.8) | 99.9  (99.8 – 99.9) | 0.93  (0.90 – 0.96) |
|  | ≥50 years | 75.5  (74.5 – 76.2) | 96.7  (96.3 – 97.1) | 13.3  (12.5 – 14.0) | 99.8  (99.7 – 99.9) | 0.93  (0.88 – 0.97) |
| **Black** | All | 90.7  (90.1 – 91.2) | 93.1  (92.5 – 93.6) | 5.6  (5.2 – 6.1) | 99.95  (99.9 – 100) | 0.96  (0.92 – 0.99) |
|  | Invasive | 89.5  (88.9 – 90.1) | 93.1  (92.5 – 93.6) | 4.9  (4.5 – 5.4) | 99.95  (99.9 – 100) | 0.96  (0.92 – 1.00) |
|  | ≥50 years | 89.3  (88.4 – 90.2) | 96.1  (95.5 – 96.6) | 11.5  (10.6 – 12.4) | 99.9  (99.9 – 100) | 0.96  (0.91 – 1.00) |
| **Other** | All | 90.0  (88.9 – 91.1) | 92.9  (91.9 – 93.8) | 4.2  (3.5 – 4.9) | 99.96  (99.9 – 100) | 0.99  (0.96 – 1.00) |
|  | Invasive | 100  (100 – 100) | 92.9  (91.9 – 93.8) | 3.3  (2.7 – 4.0) | 100  (100 – 100) | 0.99  (0.98 – 1.00) |
|  | ≥50 years | 100  (100 – 100) | 95.8  (94.8 – 96.9) | 12.1  (10.4 – 13.8) | 100  (100 – 100) | 0.99  (0.99 – 1.00) |
| **Mixed** | All | 70.0  (68.3 – 71.7) | 93.7  (92.7 – 94.6) | 3.8  (3.1 – 4.5) | 99.9  (99.8 – 100) | 0.93  (0.86 – 1.00) |
|  | Invasive | 87.5  (86.3 – 88.7) | 93.7  (92.7 – 94.6) | 3.8  (3.1 – 4.5) | 99.96  (99.9 – 100) | 0.97  (0.94 – 1.00) |
|  | ≥50 years | 100  (100 – 100) | 96.8  (95.9 – 97.8) | 8.9  (7.3 – 10.4) | 100  (100 – 100) | 0.99  (0.99 – 1.00) |
| **Unknown** | All | 87.0  (85.6 – 88.2) | 92.6  (91.5 – 93.6) | 10.1  (8.9 – 11.2) | 99.9  (99.7 – 100) | 0.95  (0.91 – 1.00) |
|  | Invasive | 94.7  (93.9 – 95.6) | 92.6  (91.5 – 93.6) | 9.1  (8.0 – 10.3) | 99.96  (99.9 – 100) | 0.98  (0.93 – 0.94) |
|  | ≥50 years | 84.2  (82.5 – 86.0) | 92.8  (91.5 – 94.0) | 11.9  (10.4 – 13.5) | 99.8  (99.6 - 100) | 0.95  (0.97 – 1.00) |
| **All** | All | 79.5  (79.3 – 79.6) | 93.7  (93.7 – 93.8) | 9.7  (9.6 – 9.8) | 99.8  (99.8 – 99.8) | 0.93  (0.92 – 0.93) |
|  | Invasive | 85.5  (85.4 – 85.6) | 93.7  (93.6 – 93.8) | 8.5  (8.5 – 8.6) | 99.9  (99.9 – 99.9) | 0.95  (0.94 – 0.95) |
|  | ≥50 years | 81.4  (81.3 – 81.6) | 94.7  (94.6 – 94.8) | 14.4  (14.3 – 14.5) | 99.8  (99.8 – 99.8) | 0.94  (0.93 – 0.94) |
