# Supplementary material for: The diagnostic performance of CA-125 for the detection of ovarian cancer in women from different ethnic groups: a cohort study of English primary care data
Source: J Ovarian Res. 2024 Aug 26;17:173. doi: 10.1186/s13048-024-01490-5 (PMC11346194; doi:10.1186/s13048-024-01490-5)
Supplement: Supplementary file 5 — Supplementary Material 5 [file 13048_2024_1490_MOESM5_ESM.docx]

|  | | | | |
| --- | --- | --- | --- | --- |
| **%** (95% CI) | **White** | **Asian** | **Black** | **All** |
| **PPV** | 10.2  (10.1 – 10.3) | 4.9  (4.6 – 5.3) | 5.6  (5.2 – 6.1) | 9.7  (9.6 – 9.8) |
| **Age- adjusted PPV** | 9.6  (9.1 – 10.1) | 7.9  (6.1 – 10.2) | 8.3  (6.0 – 11.2) | 9.4  (9.0 – 9.9) |
| **Adjustedᶧ PPV** | 9.3  (8.8 – 9.8) | 7.8  (6.1 – 10.1) | 8.4  (6.1 – 11.4) | 9.1  (8.7 – 9.6) |
| **AUC** | 0.93  (0.92 – 0.93) | 0.90  (0.86 – 0.94) | 0.96  (0.92 – 0.99) | 0.93  (0.92 – 0.93) |
| **Age-adjusted AUC** | 0.90  (0.89 – 0.91) | 0.87  (0.81 – 0.92) | 0.95  (0.92 – 0.99) | 0.90  (0.90 – 0.91) |
| **Adjustedᶧ AUC** | 0.90  (0.90 – 0.91) | 0.86  (0.81 – 0.91) | 0.95  (0.91 – 0.99) | 0.90  (0.90 – 0.91) |
| **Age-adjusted diagnostic OR** | 69.5  (62.9 – 76.8) | 57.7  (35.0 – 95.2) | 190.2  (67.5 – 536.2) | 70.3  (63.6 – 77.4) |
| **Adjustedᶧ diagnostic OR** | 70.7  (64.0 - 78.2) | 55.8  (33.8 - 92.2) | 187.4  (66.4 - 528.4) | 71.3  (64.7 – 78.6) |

**Supplementary 5:** Unadjusted, age-adjusted, and fully adjusted PPVs, AUCs and diagnostic ORs of a high CA125 result for White, Asian and Black women.

**ᶧ** Adjustments made for age group in 10-year categories, CMS, IMD, and year of test.
