# Supplementary material for: The diagnostic performance of CA-125 for the detection of ovarian cancer in women from different ethnic groups: a cohort study of English primary care data
Source: J Ovarian Res. 2024 Aug 26;17:173. doi: 10.1186/s13048-024-01490-5 (PMC11346194; doi:10.1186/s13048-024-01490-5)
Supplement: Supplementary file 6 — Supplementary Material 6 [file 13048_2024_1490_MOESM6_ESM.docx]

| **Ethnic group** | **Normal CA-125 result** | | | **High CA-125 result** | | | **Odds ratio (95% CI)** |
| --- | --- | --- | --- | --- | --- | --- | --- |
|  | **n** | **Unadjusted %** | **Adjusted % (95% CI)** | **n** | **Unadjusted %** | **Adjusted % (95% CI)** |  |
| White | 533 | 0.19 | 0.14 (0.13 to 0.16) | 2,056 | 10.22 | 9.31 (8.83 to 9.82) | 70.73 (63.95 to 78.23) |
| Asian | 23 | 0.16 | 0.15 (0.10 to 0.23) | 58 | 4.94 | 7.84 (6.06 to 10.08) | 55.83 (33.82 to 92.17) |
| Black | 4 | 0.05 | 0.05 (0.02 to 0.13) | 39 | 5.63 | 8.39 (6.12 to 11.41) | 187.36 (66.44 to 528.35) |
| Other | 1 | 0.04 | 0.04 (0.01 to 0.28) | 9 | 4.21 | 5.91 (3.07 to 11.06) | 161.49 (20.24 to 1288.29) |
| Mixed | 3 | 0.11 | 0.12 (0.04 to 0.37) | 7 | 3.8 | 6.35 (3.03 to 12.80) | 56.83 (14.43 to 223.85) |
| Unknown | 3 | 0.13 | 0.09 (0.03 to 0.29) | 20 | 10.05 | 7.40 (4.66 to 11.55) | 85.47 (24.89 to 293.53) |

Supplementary 6a: unadjusted and adjusted ovarian cancer incidence rates by CA-125 test result and ethnicity, and odds ratio of an ovarian cancer diagnosis following a raised CA-125 result, by ethnicity.

| **Ethnic group** | **Normal CA-125 result** | | | **High CA-125 result** | | | **Odds ratio (95% CI)** |
| --- | --- | --- | --- | --- | --- | --- | --- |
|  | **n** | **Unadjusted %** | **Adjusted % (95% CI)** | **n** | **Unadjusted %** | **Adjusted % (95% CI)** |  |
| White | 301 | 0.11 | 0.11 (0.10 to 0.12) | 1,789 | 9.00 | 10.09 (9.61 to 10.58) | 108.12 (95.24 to 122.73) |
| Asian | 17 | 0.12 | 0.15 (0.08 to 0.23) | 52 | 4.44 | 9.82 (7.37 to 12.26) | 76.19 (43.09 to 134.72) |
| Black | 4 | 0.05 | 0.07 (0.00 to 0.13) | 34 | 4.93 | 9.83 (6.76 to 10.58) | 172.36 (60.62 to 490.08) |
| Other | 0 | 0.00 | - | 7 | 3.30 | 6.51 (1.99 to 11.03) | - |
| Mixed | 1 | 0.04 | 0.06 (0.00 to 0.16) | 7 | 3.80 | 8.80 (2.87 to 14.72) | 184.79 (22.42 to 1523.09) |
| Unknown | 1 | 0.04 | 0.04 (0.00 to 0.12) | 18 | 9.14 | 8.50 (4.78 to 12.23) | 235.33 (31.09 to 1781.18) |

Supplementary 6b: unadjusted and adjusted incidence rates of invasive ovarian cancer by CA-125 test result and ethnicity, and odds ratio of an being diagnosed with an invasive ovarian cancer following a raised CA-125 result, by ethnicity.

| **Ethnic group** | **Normal CA-125 result** | | | **High CA-125 result** | | | **Odds ratio (95% CI)** |
| --- | --- | --- | --- | --- | --- | --- | --- |
|  | **n** | **Unadjusted %** | **Adjusted % (95% CI)** | **n** | **Unadjusted %** | **Adjusted % (95% CI)** |  |
| White | 79 | 17.87 | 34.11 (26.70-42.38) | 1174 | 67.78 | 75.90 (72.57-78.94) | 6.08 (4.13-8.96) |
| Asian | 3 | 18.75 | 28.39 (7.74-65.21) | 39 | 69.64 | 86.17 (72.33-93.69) | 15.72 (2.66-93.05) |
| Black | 2 | 66.67 | 83.47 (20.48-99.00) | 20 | 66.67 | 66.78 (41.44-85.10) | 0.40 (0.02-9.25) |
| Other | 0 | 0.00 | - | 4 | 66.67 | 66.59 (13.68-96.16) | - |
| Mixed | 0 | 0.00 | - | 2 | 28.57 | 48.40 (10.08-88.70) | - |
| Unknown | 0 | 0.00 | - | 10 | 66.67 | 62.86 (26.78-88.68) | - |

Supplementary table 6c: unadjusted and adjusted incidence of being diagnosed with ovarian cancer at an advanced stage by CA-125 test result and ethnicity, and odds ratio of being diagnosed at an advanced stage following a raised CA-125 result, by ethnicity.
