# Supplementary material for: The diagnostic performance of CA-125 for the detection of ovarian cancer in women from different ethnic groups: a cohort study of English primary care data
Source: J Ovarian Res. 2024 Aug 26;17:173. doi: 10.1186/s13048-024-01490-5 (PMC11346194; doi:10.1186/s13048-024-01490-5)
Supplement: Supplementary file 7 — Supplementary Material 7 [file 13048_2024_1490_MOESM7_ESM.docx]

**Supplementary 7: the estimated incidence of ovarian cancer by CA-125 level with 95% confidence intervals**

| **Estimated ovarian cancer incidence at 35U/ml, % (95% CI)** | | | | | | | |
| --- | --- | --- | --- | --- | --- | --- | --- |
|  | **White** | **Asian** | **Black** | **Other** | **Mixed** | **Unknown** | **All** |
| All, ≥40 years | 1.8 (1.7 - 1.9) | 0.7 (0.5 - 1.0) | 0.9 (0.5 - 1.5) | 0.6 (0.2 - 2.1) | 0.8 (0.4 - 2.0) | 1.9 (0.9 - 3.9) | 1.7 (1.6 - 1.8) |
| Invasive, ≥40 years | 1.3 (1.2 – 1.4) | 0.6 (0.3 – 0.9) | 0.6 (0.3 – 1.2) | - | 0.9 (0.3 – 2.4) | 1.7 (0.7 – 3.9) | 1.3 (1.2 – 1.4) |
| All, ≥50 years | 2.5 (2.3 – 2.7) | 1.2 (0.8 – 1.8) | 1.6 (0.9 – 2.8) | - | - | 2.1 (0.9 – 4.9) | 2.4 (2.9 – 2.6) |
| Invasive, ≥50 years | 1.9 (1.8 – 2.1) | 1.2 (0.8 – 2.0) | 1.1 (0.6 – 2.0) | - | - | 2.3 (0.9 – 5.8) | 1.9 (1.8 – 2.1) |
| **CA125 level representing an estimated 3% incidence, U/ml (95% CI)** | | | | | | | |
|  | **White** | **Asian** | **Black** | **Other** | **Mixed** | **Unknown** | **All** |
| All, ≥40 years | 54 (51 - 57) | 103 (85 - 125) | 87 (62 - 115) | 101 (43 - 175) | 99 (54 - 189) | 55 (31 - 107) | 57 (54 - 59) |
| Invasive, ≥40 years | 67 (64 - 71) | 115 (91 - 144) | 103 (74 - 138) | - | 99 (45 - 196) | 68 (28 - 130) | 70 (2.8 – 3.2) |
| All, ≥50 years | 42 (39 - 44) | 69 (52 - 89) | 57 (37 - 82) | - | - | 49 (27 - 113) | 43 (40 - 45) |
| Invasive, ≥50 years | 51 (48 - 55) | 68 (49 - 90) | 70 (49 - 98) | - | - | 49 (0 - 113) | 52 (48 - 55) |

.
