# Supplementary material for: The diagnostic performance of CA-125 for the detection of ovarian cancer in women from different ethnic groups: a cohort study of English primary care data
Source: J Ovarian Res. 2024 Aug 26;17:173. doi: 10.1186/s13048-024-01490-5 (PMC11346194; doi:10.1186/s13048-024-01490-5)
Supplement: Supplementary file 8 — Supplementary Material 8 [file 13048_2024_1490_MOESM8_ESM.docx]

| **Diagnostic accuracy of CA-125 for the detection of ovarian cancer** | | | | | | | | | |
| --- | --- | --- | --- | --- | --- | --- | --- | --- | --- |
| Threshold ≥35U/ml, **%** (95% CI) | | | | | Ethnicity-specific threshold, **%** (95% CI) | | | | |
|  | **White** | **Asian** | **Black** | **All** |  | **White** | **Asian** | **Black** | **All** |
| **Sensitivity** | 79.4  (79.3 – 79.6) | 71.6  (70.9 – 72.3) | 90.7  (90.1 – 91.2) | 79.5  (79.3 – 79.6) | **Sensitivity** | 78.7  (78.5 – 78.9) | 64.2  (63.1 – 65.2) | 67.9  (66.6 – 69.2) | 78.0  (77.8 – 78.1) |
| **Specificity** | 93.8  (93.7 – 93.9) | 92.9  (92.5 – 93.3) | 93.1  (92.5 – 93.6) | 93.7  (93.7 – 93.8) | **Specificity** | 95.8  (95.7 – 95.9) | 98.5  (98.2 – 98.7) | 98.2  (97.8 – 98.6) | 96.2  (96.1 – 96.3) |
| **PPV** | 10.2  (10.1 – 10.3) | 4.9  (4.6 – 5.3) | 5.6  (5.2 – 6.1) | 9.7  (9.6 – 9.8) | **PPV** | 17.6  (17.5 – 17.8) | 21.9  (21.0 – 22.8) | 17.8  (16.7 – 18.8) | 18.4  (18.3 – 18.6) |
| **NPV** | 99.8  (99.8 – 99.8) | 99.9  (99.8 – 99.9) | 99.95  (99.9 – 100) | 99.8  (99.8 – 99.8) | **NPV** | 99.8  (99.7 – 99.8) | 99.8  (99.7 – 99.9) | 99.8  (99.7 – 99.9) | 99.8  (99.7 – 99.8) |

Supplementary 8a: the diagnostic performance of CA-125 at the current threshold (35U/mL) and the ethnicity-specific thresholds.

|  | **White, n** | | | **Asian, n** | | | **Black, n** | | | **All, n** | | |
| --- | --- | --- | --- | --- | --- | --- | --- | --- | --- | --- | --- | --- |
| **CA-125** | **No ovarian cancer** | **Ovarian Cancer** | **Total** | **No ovarian cancer** | **Ovarian Cancer** | **Total** | **No ovarian cancer** | **Ovarian Cancer** | **Total** | **No ovarian cancer** | **Ovarian Cancer** | **Total** |
| **<35U/ml** | 184,333 | 416 | 184,749 | 7,659 | 13 | 7,672 | 4,686 | 3 | 4,689 | 200,783 | 435 | 201,218 |
| **≥35U/ml** | 10,658 | 1,812 | 12,470 | 262 | 40 | 302 | 192 | 25 | 217 | 11,329 | 1,905 | 13,234 |
| **Total** | 194,991 | 2,228 | 197,219 | 7,921 | 53 | 7,974 | 4,878 | 28 | 4,906 | 212,112 | 2,340 | 214,452 |
| **<ethnic-specific threshold** | 186,795 | 475 | 187,270 | 7,800 | 19 | 7,819 | 4,790 | 9 | 4,799 | 204,029 | 516 | 204,545 |
| **≥ethnic-specific threshold** | 8,196 | 1,753 | 9,949 | 121 | 34 | 155 | 88 | 19 | 107 | 8,083 | 1,824 | 9,907 |
| **Total** | 194,991 | 2,228 | 197,219 | 7,921 | 53 | 7,974 | 4,878 | 28 | 4,906 | 212,112 | 2,340 | 214,452 |

Supplementary 8b: 2x2 table outlining true negatives, false negatives, true positives, and false positives.
